# Sleep Disruption and Daytime Sleepiness Correlating with Disease Severity and Insulin Resistance in Non-Alcoholic Fatty Liver Disease: A Comparison with Healthy Controls

**DOI:** 10.1371/journal.pone.0143293

**Published:** 2015-11-17

**Authors:** Christine Bernsmeier, Diego M. Weisskopf, Marlon O. Pflueger, Jan Mosimann, Benedetta Campana, Luigi Terracciano, Christoph Beglinger, Markus H. Heim, Christian Cajochen

**Affiliations:** 1 Division of Gastroenterology and Hepatology, University Hospital Basel, Basel, Switzerland; 2 Department of Biomedicine, University Hospital Basel, Basel, Switzerland; 3 Centre for Chronobiology, Psychiatric Hospital of the University of Basel, Basel, Switzerland; 4 Forensic Psychiatric Clinic, Psychiatric Hospital of the University of Basel, Basel, Switzerland; 5 Institute of Pathology, University Hospital Basel, Basel, Switzerland; Institute of Hepatology, Foundation for Liver Research, UNITED KINGDOM

## Abstract

**Background & Aims:**

Sleep disturbance is associated with the development of obesity, diabetes and hepatic steatosis in murine models. Hepatic triglyceride accumulation oscillates in a circadian rhythm regulated by clock genes, light-dark cycle and feeding time in mice. The role of the sleep-wake cycle in the pathogenesis of human non-alcoholic fatty liver disease (NAFLD) is indeterminate. We sought to detail sleep characteristics, daytime sleepiness and meal times in relation to disease severity in patients with NAFLD.

**Methods:**

Basic Sleep duration and latency, daytime sleepiness (Epworth sleepiness scale), Pittsburgh sleep quality index, positive and negative affect scale, Munich Chronotype Questionnaire and an eating habit questionnaire were assessed in 46 patients with biopsy-proven NAFLD and 22 healthy controls, and correlated with biochemical and histological parameters.

**Results:**

In NAFLD compared to healthy controls, time to fall asleep was vastly prolonged (26.9 vs. 9.8 min., p = 0.0176) and sleep duration was shortened (6.3 vs. 7.2 hours, p = 0.0149). Sleep quality was poor (Pittsburgh sleep quality index 8.2 vs. 4.7, p = 0.0074) and correlated with changes in affect. Meal frequency was shifted towards night-times (p = 0.001). In NAFLD but not controls, daytime sleepiness significantly correlated with liver enzymes (ALAT [r = 0.44, p = 0.0029], ASAT [r = 0.46, p = 0.0017]) and insulin resistance (HOMA-IR [r = 0.5, p = 0.0009]) independent of cirrhosis. In patients with fibrosis, daytime sleepiness correlated with the degree of fibrosis (r = 0.364, p = 0.019).

**Conclusions:**

In NAFLD sleep duration was shortened, sleep onset was delayed and sleep quality poor. Food-intake was shifted towards the night. Daytime sleepiness was positively linked to biochemical and histologic surrogates of disease severity. The data may indicate a role for sleep-wake cycle regulation and timing of food-intake in the pathogenesis of human NAFLD as suggested from murine models.

## Introduction

Non-alcoholic fatty liver disease (NAFLD) has become one of the most prevalent chronic liver diseases. It is perceived as both cause and consequence of the metabolic syndrome, and involves various liver-related and unrelated conditions such as cirrhosis, end-stage liver disease, hepatocellular carcinoma, cardiovascular disease and diabetes [1]. The raising incidence over the previous decades also infers common aetiologies as key-effectors in its pathogenesis. Obesity, mal-nutrition and physical inactivity are recognised as such key-effectors. Also genetic variants such as an allele in PNPLA3 have been shown to play a role [2]. Sleep disruption is another possibly under-appreciated condition having evolved with industrialism, that has been associated with metabolic disease in humans [3,4].

The circadian clock is indispensable for the regulation of various metabolic pathways and other physiologic mechanisms. A central so-called “master” circadian pacemaker is located in the hypothalamus, and is synchronising peripheral clocks present in all mammalian cells [5]. In hepatocytes, peripheral clocks orchestrate many genes regulating nutrient sensing, storage and release in cooperation with acute regulatory pathways [6,7]. Sleep disruption is associated with the development of obesity and diabetes in murine models and humans. The underlying mechanisms are complex and are supposed to be regulated through both gene regulation as well as light/dark cycles in a bi-directionally manner [7,8].

The association of clock-gene disruption and hepatic steatosis has been shown in different murine models. Homozygous Clock mutant mice were hyperphagic, obese and developed hyperglycaemia, hyperlipidaemia and steatosis [9]. Also mice deficient in Bmal1, another important clock gene, accumulated fat in the liver [10]. Vice versa high-calorie diet altered the function of the mammalian circadian clock [11]. Recently, hepatic triglyceride accumulation was shown to oscillate in a circadian rhythm, dually regulated by clock genes and feeding time in wild-type and clock-disrupted mice [12]. In particular, in wild-type mice restriction to night-time feeding reduced hepatic triglyceride accumulation by 50% [12].

Circadian rhythm disruption has been shown to disturb the glucose-insulin metabolism, appetite and related hormones also in humans [3,4,13,14]. An analysis of the human metabolome found that 15% of all plasma metabolites were clock regulated, a high proportion of these metabolites were fatty acids [15].

In human NAFLD fatigue has been observed independently of cirrhosis and has previously been correlated to daytime sleepiness but not to biochemical or histologic markers of disease severity or insulin resistance (IR) [16]. Recently, impaired sleep duration and sleep quality in general have been associated with a risk for NAFLD in the middle-aged population [17]. A pilot study, evaluating the use of the circadian hormone melatonin as an antioxidant for the treatment of NAFLD demonstrated beneficial effects on liver enzymes [18,19].

Despite the growing data in rodents [9–12] the role of sleep-wake cycle regulation in the pathogenesis of NAFLD in humans remains insufficiently understood. Here, we systematically delineate quantitative and qualitative characteristics of sleep, food intake dynamics and daytime sleepiness in patients with NAFLD in their relation to biochemical and histologic markers of disease severity.

## Patients and Methods

### Ethics Statement

The protocol conformed to ethical guidelines of the 1975 Declaration of Helsinki and was approved by the ethics committee of the Canton Basel (Ethikkommission beider Basel). Written informed consent was obtained from all participants.

### Patients

The cohort included 46 patients with biopsy proven NAFLD, including patients with simple steatosis (n = 12) or non-alcoholic steatohepatitis (NASH; n = 34) and 22 healthy controls. Patients were participants of the Basel NAFLD cohort who consented to participate in evaluation of their sleep behaviour. The Basel NAFLD cohort is a prospective observational study including 89 patients with biopsy proven NAFLD at the time the sleep assessment study was carried out. All patients have regular yearly follow-up visits, and were asked to participate; 46 out of 89 patients (51%) consented to the sleep assessment sub-study in July 2012. Liver biopsies had previously been obtained at inclusion into the cohort study and evaluated by a single hepato-histopathologist (L.T.). The median time of biopsy before assessment of sleep behaviour was 4 years. Histologic differentiation between simple steatosis and NASH, grading and staging of disease, had been assessed using the NAFLD activity score (NAS), defining NASH if NAS was ≥4 and histological features of steatohepatitis were described [20,21]. Exclusion criteria for NAFLD patients were alcohol consumption >40g/d for men and >20g/d for women and concomitant liver disease.

Healthy controls were screened for the metabolic syndrome and liver disease by medical history and a blood sample including liver enzymes (ASAT, ALAT, GGT), fasting glucose and lipids. Exclusion criteria for controls were alcohol consumption (criteria stated above), smoking, any liver disease, elevated liver enzymes, elevated lipids, IR or intake of any drug with a known influence on glucose homeostasis.

### Questionnaires

The below cited questionnaires were completed by the study participants independently, without any support by the investigators. Questionnaires were provided in the patient’s native language whenever possible and asked to be returned within 4 to max. 12 weeks to avoid seasonal influences on the sleep rhythm (from July to September 2012).

The Pittsburgh sleep quality index (PSQI) [22], daytime sleepiness (Epworth sleepiness scale [ESS] [23]), the positive and negative affect scale (PANAS [24]) and the Munich Chronotype Questionnaire (MCTQ) [25] have been assessed. The eating habit questionnaire was assembled to assess eating frequency, timing and quality in parts referring to Nutricalc questionnaire, Swiss society of nutrition (S1 File).

### Hormones, glucose and liver enzymes

Blood glucose concentrations were measured using hexokinase-method (Roche, Basel, Switzerland). Insulin was assessed by human insulin ELISA, Merck Millipore, Billerica, USA; sensitivity of the assay 1 μU/mL. Liver enzymes were assessed by routine diagnostics by standardized International Federation of Clinical Chemistry protocol using pyridoxale-5phosphate.

### Insulin resistance

IR was assessed calculating the homeostasis model assessment HOMA-IR [26].

### Oral glucose tolerance test

Oral glucose tolerance test (oGTT) was performed after an overnight fast according to a standardized protocol using 75g of glucose in 300 ml tap water (300 kcal) as previously described[27]. Plasma samples were stored at −70°C for subsequent assessment of fasting insulin and baseline liver enzymes.

### Statistical analysis

Statistical analysis was done using R studio software version 0.97.551, Boston, USA and R environment for statistical computing (2014), R Foundation for Statistical Computing, Vienna, Austria. Graphs were drawn using GraphPad Prism 6.0c for Macintosh, San Diego, USA.

Numbers of analysed values are specified for each variable. Data were presented as means ± SEM or median (IQR). For data that did not follow a normal distribution, the significance of differences was tested using the Mann-Whitney test. Contingency tables were analysed using Chi-square test. Spearman’s correlations were calculated, and differences and correlations were considered significant at a level of p<0.05.

In order to identify predictors of sleep duration a model validation has been calculated. The technique assesses how the statistical results of this cohort will generalise to an independent data set. We defined the saturated model including the following possible predictors of sleep duration: ESS, PSQI, Age, BMI, ASAT, ALAT, GGT, fasting glucose, fasting insulin, HOMA, metabolic syndrome and NAS. Interaction with the groups (controls vs. NAFLD patients) was specified. Terms with high variance inflation factor (VIF) were deleted. The following model remained: sleep duration ~ ESS + age + BMI + ASAT + ALAT + GGT + fasting glucose + metabolic syndrome + NAS + age:group + group:insulin. The variables were sorted according to effect sizes and eliminated using cross-validation (Jack-Knife/leave-one-out). The model with the smallest prediction error was identified as the model predicting sleep duration best: lm(formula = sleep duration ~ scale(ESS) + group:scale(insulin) + scale(BMI) + scale(ASAT), data = feed).

For the comparison of meal frequency and times between the groups, a generalized linear mixed model (GLMM) was carried out using a binomial distribution and a logit-link function. The occurrence of a meal during one of seven daytime intervals (time) was dichotomously coded and served as dependent variable. Time was coded as polynomial up to the order of six. A random intercept was modelled by subject. The result indicated meal frequency as function of time as polynomial of order one, two, four and six. The data were modelled by the R environment for statistical computing (2014), R Foundation for Statistical Computing, Vienna, Austria.

## Results

### Characteristics of NAFLD patients

NAFLD patients showed clinical and biochemical characteristics of disease: Weight and BMI were significantly higher compared to controls. Fasting insulin and HOMA-IR were significantly higher, while fasting glucose did not significantly differ (Table 1). Diabetes was present in 10 patients (20.4%). Liver enzymes (ASAT, ALAT, GGT) were significantly elevated in NAFLD compared to controls. Mean NAS score was 2.5 for patients with simple steatosis and 5.1 for NASH; mean fibrosis scores were 0.3 and 2.2 respectively (Table 1). Cirrhosis was present in 9 patients (18.4%).

**Table 1:** Biochemical parameters, histological features and sleep characteristics of patients with non-alcoholic fatty liver disease (NAFLD) and controls.

	NAFLD						Controls		NAFLD vs. Controls
	All		Steatosis		NASH				
	mean±SEM	median(IQR)	mean±SEM	median(IQR)	mean±SEM	median(IQR)	mean±SEM	median(IQR)	
Age (years)	52.5±2.0	54.5 (19.25)	49.2 ± 3.1	50.0 (15.0)	53.6 ± 2.5	56.0 (23.8)	45.0±3.0	49.0 (21.5)	*ns*
Weight (kg)	88.4±2.3	90.0 (21.1)	80.6 ± 2.5	79.5 (14.9)	91.2 ± 2.7	93.9 (25.6)	71.2±1.9	71.0 (11.7)	*p<0*.*0001*
BMI (kg/m^2^)	29.8±0.7	29.5 (6.7)	27.9 ± 0.8	28.9 (6.0)	30.5 ± 0.9	30.8 (8.3)	23.1 ± 0.4	23.1 (3.1)	*p<0*.*0001*
Fasting glucose (mmol/l)	6.1 ± 0.4	5.2 (1.5)	5.1 ± 0.2	5.0 (0.825)	6.5 ± 0.5	5.4 (2.4)	5.2 ± 0.1	5.3 (3.8)	*ns*
Fasting insulin (mU/l)	12.6 ± 1.9	8.0 (11.8)	5.4 ± 0.6	5.9 (2.6)	14.9 ± 2.3	10.6 (13.9)	5.0 ± 0.4	4.5±3.3	*p = 0*.*0026*
HOMA-IR	3.8 ± 0.7	1.8 (3.8)	1.2 ± 0.1	1.3 (0.6)	4.6 ± 0.9	2.4 (4.4)	1.3 ± 0.1	1.0±0.9	*p = 0*.*0030*
ASAT (U/l)	46.9 ± 4.7	38.0 (28.5)	33.2 ± 3.1	32.0 (18.0)	51.4 ± 6.0	40.0 (32.8)	26.2 ± 1.2	26.0±7.8	*p = 0*.*0004*
ALAT (U/l)	62.2 ± 4.8	52.0 (43.0)	52.7 ± 7.6	41.0 (43.0)	65.2 ± 5.7	58.0 (42.5)	22.0 ± 1.7	21.5±10.3	*p<0*.*0001*
GGT (U/l)	101.6 ±16.5	63.0 (70.0)	60.9 ± 9.3	58.0 (52.0)	114.7 ± 21.2	66.5 (92.1)	26.0 ± 4.5	18.0±21.5	*p<0*.*0001*
NAS (0–8)	4.4±0.3	4.0 (2.5)	2.5 ± 0.4	3.0 (2.0)	5.1 ± 0.3	5.0 (2.0)	-	-	*-*
Fibrosis Score (0–4)	1.7±0.2	1.0 (2.0)	0.3 ± 0.1	0.0 (0.8)	2.2 ± 0.2	2.0 (2.6)	-	-	*-*
Mallory's Hyaline (0–2)	0.2±0.1	0.0 (0.0)	0.0 ± 0.0	0.0 (0.0)	0.3 ± 0.1	0.0 (0.0)	-	-	*-*
Sleep duration (h)	6.3±0.2	6.5 (1.5)	6.3±0.4	6.0 (2.5) ‡	6.4±0.3	7.0 (1.4) §	7.2±0.2	7.0 (0.6)	*p = 0*.*0149*
Sleep latency (min.)	26.9±5.0	14.0 (38.5)	18.45±6.4	20.0 (15.0)	31.0±6.6	20.0 (40.0) §	9.8±2.0	7.5 (11.5)	*p = 0*.*0176*
Bed time (h/24h)	23.0±0.2	23.0 (1.6)	23.2±0.3	23.1 (1.8)	22.9±0.3	23.0 (1.7)	22.9±0.1	23.0 (0.9)	*ns*
MCTQ	4.7±0.4	4.6 (1.7)	4.4±0.3	4.1 (1.5)	4.9±0.5	4.7 (1.9)	4.9 ± 0.5	4.2 (1.4)	*ns*
PSQI	8.2±0.8	7.5 (8.0)	6.3±1.2	6.0 (7.0)	9.0±1.0	9.0 (7.5) §	4.7±0.8	5.0 (5.0)	*p = 0*.*0074*
PANAS-NA	19.3±1.1	17.0 (11.0)	16.3±2.0	14.5 (5.8)	20.2±1.3	18.0 (11.0) §	15.1±1.1	13.0 (11.0)	*p = 0*.*0163*
PANAS-PA	32.6±1.0	34.0 (8.0)	35.4±1.5	35.5 (9.8)	31.8±1.2	34.0 (10.0)	33.0±1.4	33.0 (7.0)	*ns*
ESS	6.8±0.7	6.0 (6.5)	5.6 ± 0.9	5.0 (5.0)	7.2±0.8	7.0 (8.0)	7.1±0.8	8.0 (6.25)	*ns*

NAFLD Patients (n = 46; simple steatosis n = 12, Non-alcoholic steatohepatitis [NASH] n = 34) and controls n = 22.

‡, significant difference Controls vs. Steatosis

§, significant difference Controls vs. NASH

Homeostasis model assessment of insulin resistance (HOMA-IR). NAFLD activity score (NAS). Hours (h). Minutes (min.). Munich Chronotype Questionnaire (MCTQ). Pittsburgh Sleep Quality Index (PSQI); a score ≤ 5 is considered good sleep quality. Positive and negative affect scale (PANAS). Epworth Sleepiness scale (ESS); a score <10 is considered normal.

### Patients with NALFD need more time to fall asleep and sleep shorter than controls

**Fig 1:**
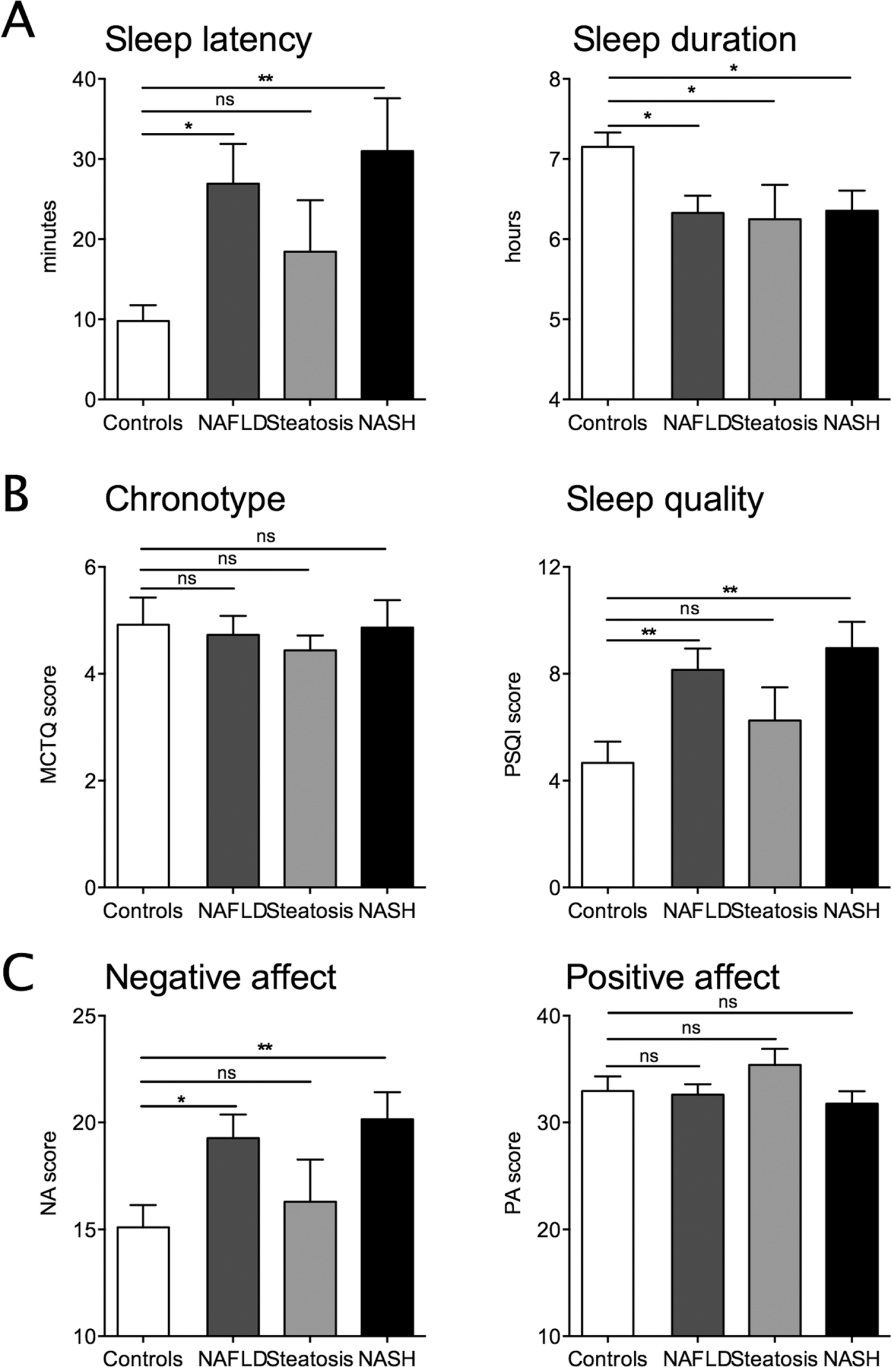
Sleep characteristics detailed in patients with Non-alcoholic fatty liver disease (NAFLD) and controls. (A) Sleep latency (minutes; left) was significantly prolonged in NAFLD compared to controls; sleep duration was significantly reduced (hours; right). (B) The chronotype did not significantly differ between NAFLD and controls, Munich Chronotype Questionnaire (MCTQ; left) and sleep quality was poor (Pittsburgh Sleep Quality Index [PSQI]; right). A score ≤ 5 is considered good sleep quality. (C) Positive and negative affect scale (PANAS): Negative affect was significantly higher in patients (left). Positive affect did not differ (right). Controls (n = 22), NAFLD (n = 46; simple steatosis [n = 12]; Non-alcoholic steatohepatitis [NASH, n = 34]). *, p<0.05; **, p<0.001.

Time to fall asleep, so-called sleep latency, was assessed using the MCTQ. It took NAFLD patients about 3-times longer to fall asleep (mean 26.9 vs. 9.8 min., p = 0.0176) compared to controls. Sleep latency was significantly prolonged in the NASH subgroup compared to controls, but not the simple steatosis subgroup (Fig 1A/ Table 1). Sleep duration was assessed using data derived from the PSQI. In addition to falling asleep later, NAFLD patients slept significantly shorter than controls (mean 6.3 vs. 7.2 hours, p = 0.0149); this difference was significant for both the simple steatosis- and NASH subgroups (Fig 1A/ Table 1). Sleep duration negatively correlated with weight and BMI in NAFLD but not controls (S1 Fig). Bed-time and the chronotype assessed by the Munich Chronotype Questionnaire (MCTQ) were not different between NAFLD and controls (Fig 1B/ Table 1).

In a model validation, shortened sleep duration was predictable by daytime sleepiness (ESS), BMI and ASAT, whereas prolonged sleep duration was predictable by fasting insulin with differential effect for NAFLD patients vs. controls, respectively (S1 Table).

In order to exclude a potential age-related impact on sleep-wake rhythm, we added a sub-group analysis excluding patients aged above 66 years. In this subgroup analysis age did not differ between NAFLD and controls while bodyweight, BMI, insulin resistance and liver enzymes were significantly different (S2 Table). Sleep latency was longer and sleep duration was significantly shorter in NAFLD, while MCTQ did not differ (S3 Table) as described for the entire cohort. The data suggests a disease specific sleep-wake pattern in patients with NAFLD.

### Food intake in NAFLD patients is shifted towards night times

**Fig 2:**
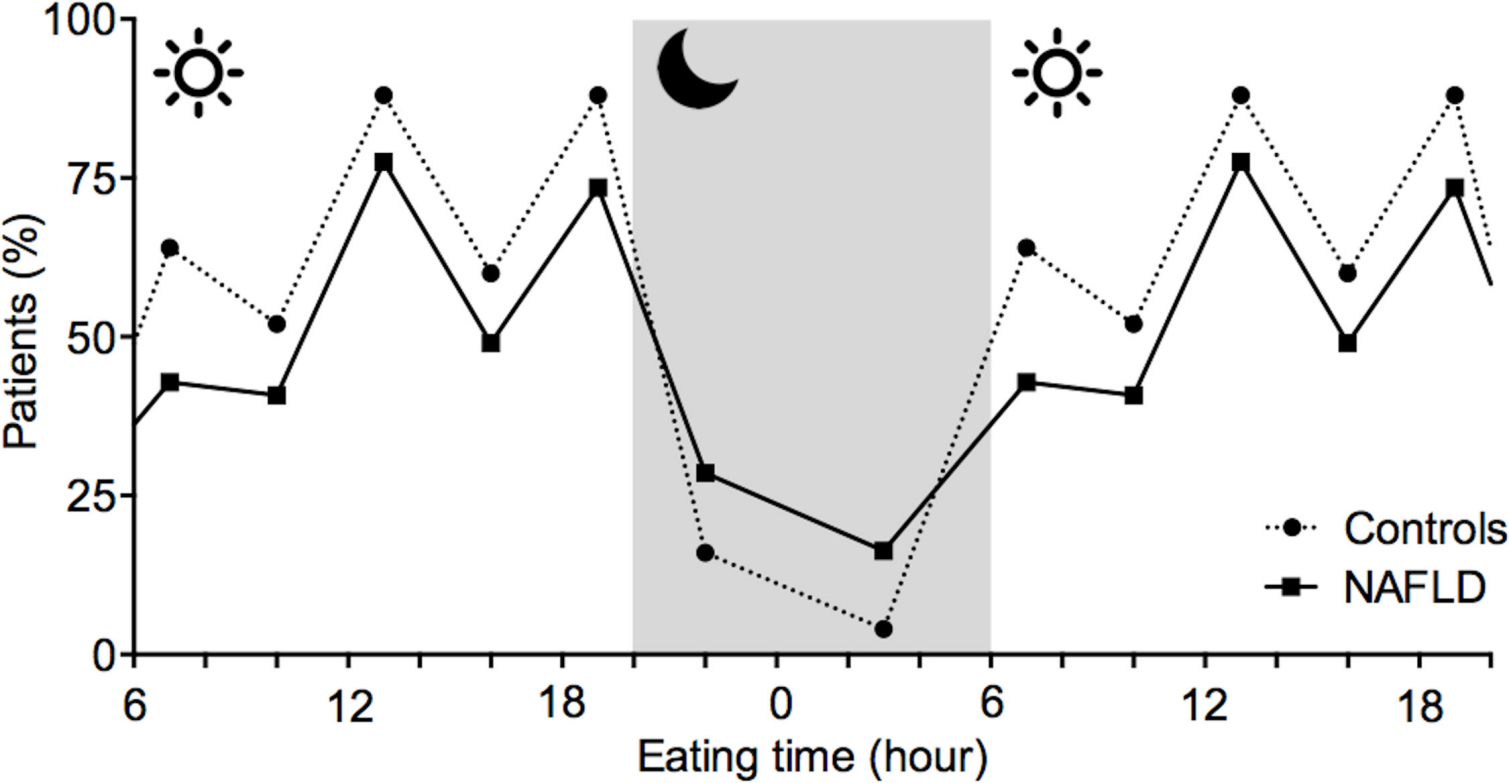
Timing of food-intake differs between NAFLD patients and controls. The percentage of patients vs. controls having meals at different times during the day and night. Compared to controls meal times in NAFLD patients were shifted towards the night (p = 0.001, generalised linear mixed model). Controls (n = 22); NAFLD (n = 44).

If NAFLD patients fall asleep later and sleep shorter, we next questioned whether also the time of food intake was changed in parallel. Indeed, nocturnal meals were numerically more frequent in NAFLD (S4 Table). In order to integratively compare meal frequency and timing of meals between NAFLD and controls over a period of 24h, we looked at frequency of meal-intake at seven daytime intervals and carried out a GLMM for statistical analysis. There was no group main effect, which indicated a balanced meal frequency across both groups. A statistically significant group x time interaction indicated a shift of meal frequency towards night times in NAFLD patients (p = 0.001) (Fig 2).

As nutritional factors possibly influencing the sleep-wake cycle, coffee consumption was significantly higher in patients compared to controls (2.4 vs. 1.7 cups/day, p = 0.0016); alcohol consumption however was significantly lower (1.4 vs. 2.0 drinks/day, p = 0.0037). The awareness for health and nutrition was significantly lower in NAFLD patients and physiological, hunger guided food intake was significantly less frequent. We did not find a significant difference in stress related food intake. In regards to physical activity, recreational physical activity was lower in NAFLD patients than in controls (S4 Table).

### Sleep quality is poor and parallels change in affect in patients with NAFLD

In addition to quantitative sleep characteristics, we evaluated sleep quality, which was poor in NAFLD patients and was significantly lower compared to controls (mean 8.2 vs. 4.7, p = 0.0074). Using the PSQI, a score > 5 is considered poor sleep quality. Subgroup analysis showed sleep quality was poorer in NASH compared to controls, but not the simple steatosis subgroup (Fig 1B/ Table 1).

Impaired sleep quality can impact on affect. In patients with NAFLD negative affect, assessed by the positive and negative affect scale (PANAS), was significantly higher compared to controls. The subgroup analysis showed a significant difference for patients with NASH but not simple steatosis. Positive affect did not differ significantly between the groups (Fig 1C/ Table 1). The same results are noticed in the subgroup analysis excluding subjects above age of 66 years (S3 Table).

To further detail the consequences of poor sleep quality on affect, we correlated the sleep quality index (PSQI) with the positive- and negative affect scale (PANAS). PSQI was negatively correlated with the positive affect scale (PANAS-PA; S2A Fig) and positively correlated with the negative affect scale (PANAS-NA; S2B Fig) in NAFLD patients but not controls. In NAFLD positive affect (PANAS-PA) itself was not only negatively correlated to PSQI but also to ASAT and ALAT (S2C and S2D Fig).

Sleep apnoea (OSA) is a condition frequently observed in obese patients and has been described as a risk factor for NAFLD [28]. Assessed by the PSQI, in our cohort 54% of NAFLD patients slept in the room with a partner, and 13% of these partners reported pauses in breathing at a frequency of >3 times per week. This corresponds to 7% of the entire NAFLD cohort studied. None of the healthy controls (0%), which slept in the room with a partner, reported pauses in breathing. The aetiologies of sleep disruption in NAFLD patients therefore seem to be diverse. We subsequently focussed on description of associations of sleep disruption with its consequences and disease severity of NAFLD.

### Daytime sleepiness is associated with elevated liver enzymes, insulin resistance and histologic features of NAFLD

**Fig 3:**
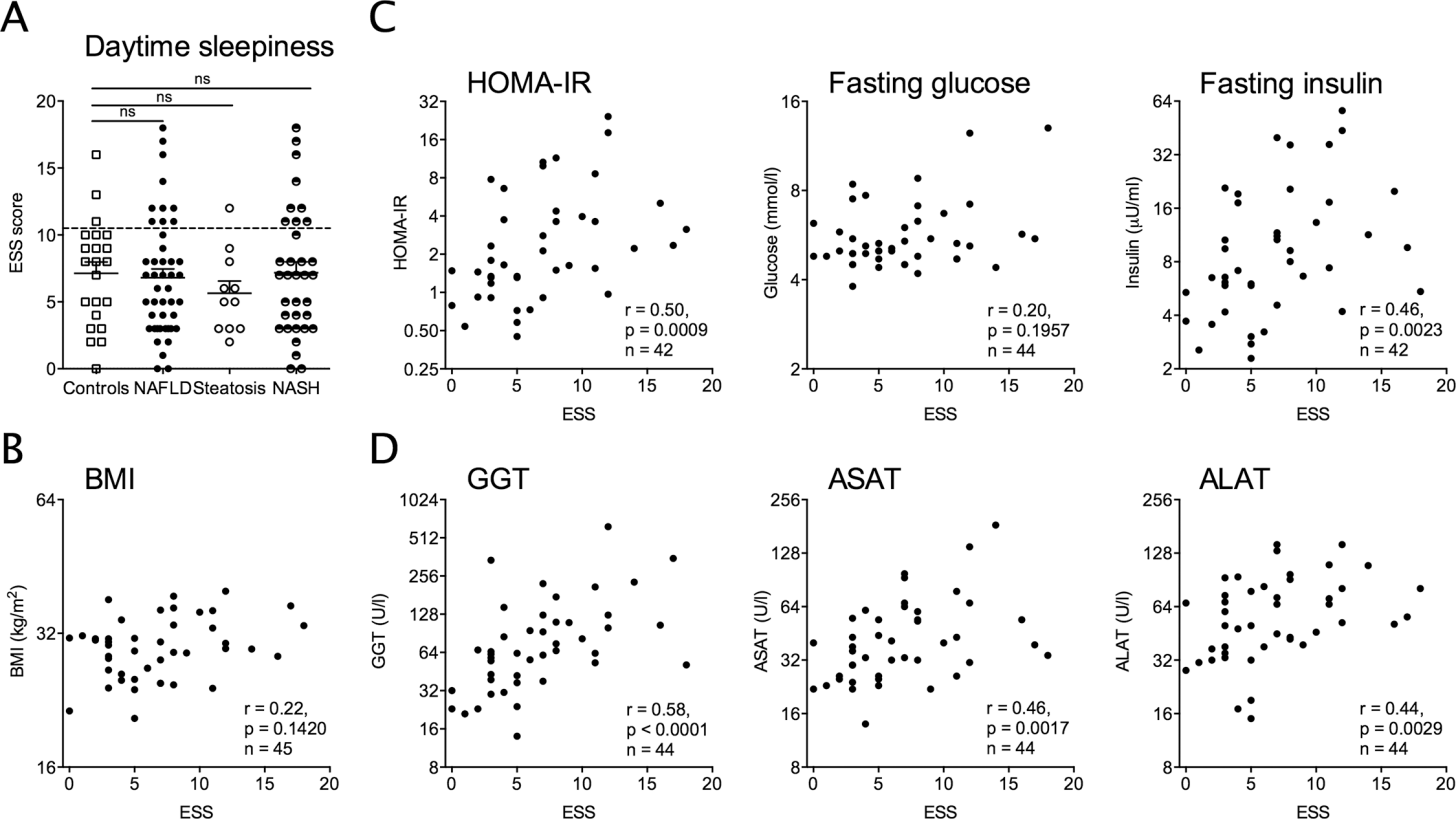
Daytime sleepiness in patients with Non-alcoholic fatty liver disease (NAFLD) is linked to insulin resistance and elevated liver enzymes. (A) Daytime sleepiness, assessed by the Epworth Sleepiness scale (ESS) was observed more often in NAFLD but median scales did not differ significantly from controls. An ESS score ≤10 is considered normal. (B-D) Daytime sleepiness is positively correlated with clinical and biochemical parameters in patients with NAFLD/NASH. Graphs showing ESS correlations with (B) BMI (kg/m^2^), (C) parameters of insulin resistance: HOMA-IR, glucose (mmol/l), insulin (μU/ml) and (D) liver enzymes: Gamma-glutamyl transpeptidase (GGT, U/l), Aspartate aminotransferase (ASAT, U/l) and Alanine aminotransferase (ALAT, U/l). Spearman correlations.

Having documented significant changes of sleep onset, sleep duration and sleep quality, the level of daytime sleepiness, a possible consequence of sleep disruption, required assessment. Using the Epworth sleepiness scale, in NAFLD patients daytime sleepiness was more often pathologic compared to controls (22.2% vs. 13.6%; ns; a scale ≤10 is considered normal), especially in the NASH subgroup (26.5% vs. 13.6%; p = 0.0228, Chi-square tests). However, the median ESS score was not significantly different between the groups (Fig 3A).

Remarkably, in NAFLD patients but not controls the scale of daytime sleepiness was correlated with various clinical factors associated with NAFLD and the metabolic syndrome. ESS was positively correlated with fasting insulin, HOMA-IR and liver enzymes: GGT, ASAT and ALAT (Fig 3B–3D).

As daytime sleepiness is a frequent clinical symptom in patients with cirrhosis, we verified these findings in the non-cirrhotic subpopulation. Interestingly, non-cirrhotic NAFLD patients showed similar characteristics of sleep disturbance as the entire cohort (low sleep quality (PSQI), high negative affect (PANAS-NA), similar chronotype (MCTQ) and daytime sleepiness (ESS) (data not shown). The sub-population equally showed a positive correlation of daytime sleepiness with fasting insulin, HOMA and liver enzymes. Also blood glucose levels following an oral glucose tolerance test (oGTT) correlated positively with the ESS scale (S5 Table).

**Fig 4:**
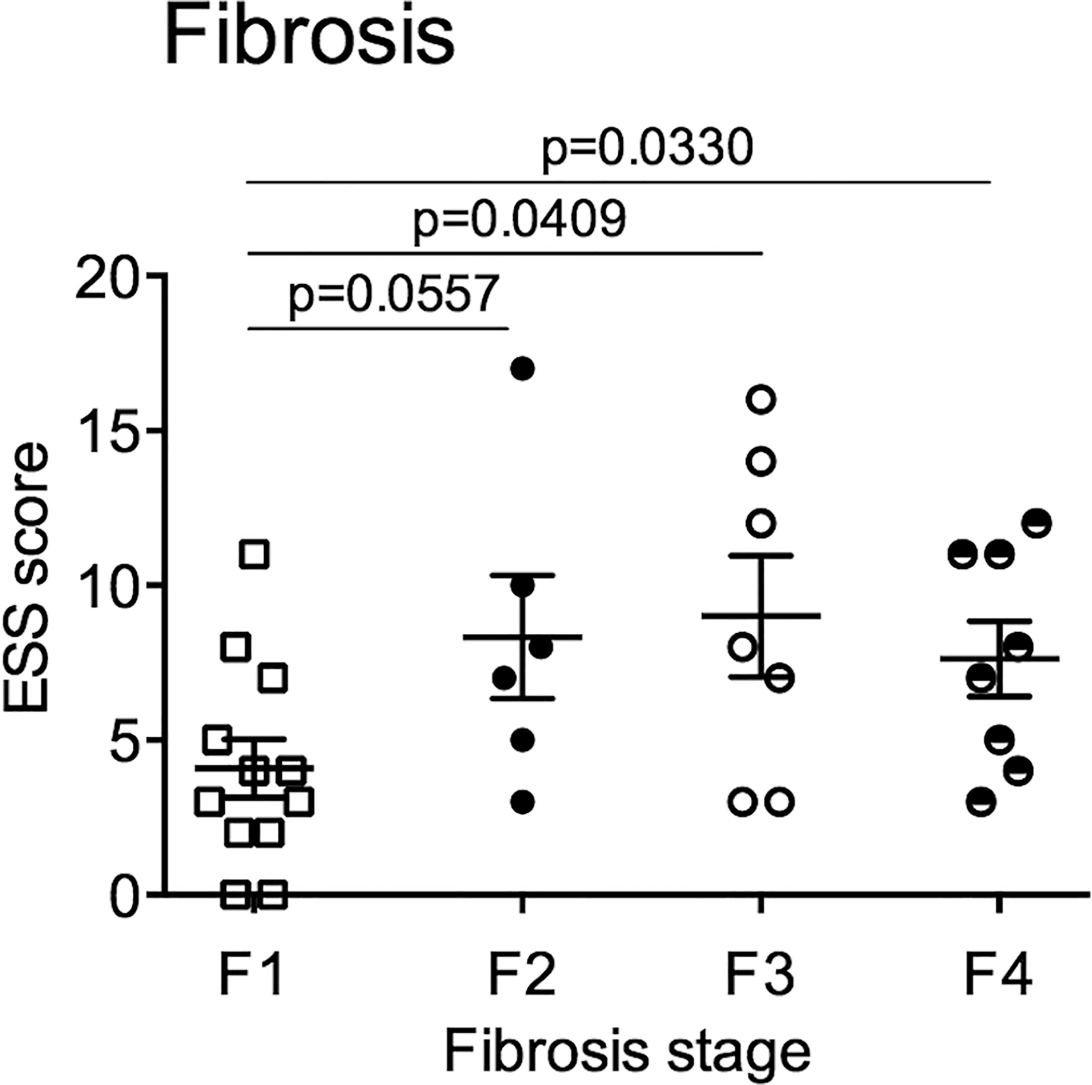
Daytime sleepiness is higher in patients with advanced fibrosis. Daytime sleepiness, assessed by the Epworth Sleepiness scale (ESS) was plotted against the stage fibrosis (NAFLD activity score) in patients with fibrosis (F1-F4). *, p<0.05.

Daytime sleepiness was furthermore associated with histological parameters of disease, namely fibrosis (Fig 4). Overall, ESS did not correlate with stage of fibrosis (Spearman’s r = 0.2047, p = 0.1774). In patients who had developed fibrosis (F1-F4) ESS was however positively correlated with the degree of fibrosis (r = 0.40; p = 0.019; n = 33). Again, the positive correlation endured if patients with established cirrhosis were excluded from the analysis (F1-F3; r = 0.47; p = 0.017; n = 25). In summary these data are suggestive for a potential role of sleep disruption in the pathogenesis of NAFLD.

## Discussion

The aims of this study were to detail quantitative and qualitative characteristics of sleep, meal times, daytime sleepiness and affect and their potential association with clinical surrogates of disease severity in patients with biopsy proven NAFLD. Further to discuss the possible significance of our data in respect to a role of sleep-wake cycle in the pathogenesis of NAFLD.

Our data were suggestive for sleep disruption in patients with NAFLD: sleep duration was shortened, time to fall asleep was prolonged, and sleep quality was poor. We identified daytime sleepiness, BMI and ASAT as predictors of shortened sleep duration, suggesting that not only obesity alone but also NAFLD itself may be linked to sleep disturbances.

Interestingly, also the timing of food-intake was shifted in NAFLD patients compared to controls, such that NAFLD patients consumed more nocturnal meals. Daytime sleepiness was more frequent in NAFLD and significantly correlated with insulin resistance, liver enzymes and hepatic fibrosis.

If supported by further studies, our findings may corroborate a pathophysiologic role of sleep disruption and a shift of food-intake towards the night in human NAFLD as proposed from data obtained in murine models. In mice, it had previously been shown that circadian rhythm gene regulation importantly impact on hepatic triglyceride storage [9,29,30], and it has been postulated that the hepatic diurnal metabolism is regulated in a reciprocal interaction of gene regulation, light/dark cycle and feeding time [7,8,11,12].

In contrast to rodents, the role of sleep in the pathophysiology of human NAFLD has not been systematically evaluated. In order to screen for potential disturbances in quantitative and qualitative characteristics of sleep we chose to initially use a selection of approved questionnaires [22–25] that have been applied for this purpose in various other clinical disorders. However, questionnaires are subjective measures that may lead to bias. Moreover, the responses may underlie seasonal variation, which we tried to avoid using data returned within a period of 3 months only. A limitation of this study was, that we opted not to include objective measures such as actigraphy, a sensor worn at the wrist that measures gross motor activity in a subject, oscillation in physiological variables during sleep, and hormones such as melatonin and cortisol. These variables need to be assessed in a well-defined cohort of NAFLD patients to validate the initial findings reported here.

Although clinically relevant, from an ethical point of view, it will be difficult to directly verify the concept postulated from rodent models in patients with NAFLD. In humans diurnal regulation of plasma lipids has been shown [15] as well as disturbance of the diurnal pattern of de-novo lipogenesis in the liver of NAFLD patients [31]. Having used a non-invasive, non-interventional approach, we delineated sleep disturbances and “feeding times” in patients with biopsy proven NAFLD. Our observations based on questionnaire method do not allow postulating a causal pathophysiologic relationship in human NAFLD yet. However, evaluating this data in reflection of the murine concept, we may hypothesize that sleep disruption including delayed onset and shortened duration as well as shifted food-intake might increase hepatic triglyceride accumulation also in humans and merit further investigation.

Moreover, the data of this study clearly link daytime sleepiness to clinical surrogates of disease severity of NAFLD (liver enzymes and fibrosis) and the metabolic syndrome (IR). The newly described close correlations of daytime sleepiness- and affect scales with biochemical parameters in patients with NAFLD were unexpected and striking. To our knowledge, there is only one report suggesting a correlation of sleep disruption and liver enzymes: In a rat model REM sleep deprivation led to increased levels of liver enzymes and pro-inflammatory cytokines in relation to the extent of sleep loss [32]. Ideally, liver enzymes and sleep parameters would have been assessed the same day; here per protocol sleep parameters were assessed during a period of three months and compared to biochemical parameters taken within the preceding year. This approach was chosen to avoid seasonal variation in sleep habits, but may limit the validity of the reported correlation. The association between daytime sleepiness and fibrosis is interesting, although we have to consider some limitation of the data. First, a certain dynamic in fibrosis evolution is expected in a proportion of the patients, as for ethical considerations we did not re-biopsy NAFLD patients at the time of sleep assessment. A recent study reported any degree of fibrosis progression in 42% of NAFLD patients, while in 58% of patients fibrosis did not progress or in contrast regressed after a median follow-up of 6.6 years [33]. However a meta-analysis of 11 cohort studies analysing fibrosis in paired biopsies of patients with NAFLD reported an average progression of 1 stage over 7.7 years only [34]. Therefore the expected fibrosis progression rate over 4 years is less than 1 fibrosis stage. Second, we observed a correlation of daytime sleepiness with stage of fibrosis in the subgroup of patients who had developed fibrosis (at least F1) only. The variation of ESS in the group of patients without any development of fibrosis (F0) may possibly be due to other influential factors in very mild NAFLD. Also, the staging of fibrosis is subject to possible sampling errors and to subjective assessment and may therefore not be an ideal parameter for correlation.

It does not surprise, that the subgroup analysis–differentiating between simple steatosis and NASH–shows significant differences for NASH, the more severe condition, only. A different explanation might be that sleep disruption impacts not only on metabolism in the liver but also on the inflammatory response. The metabolic syndrome is characterised by low-grade chronic inflammation, as is NASH. A reciprocal regulation of sleep and cytokine production by different immune effector cells has been proposed [35]. Thus, it is possible that sleep disruption promotes hepatic inflammation in NAFLD, i.e. steatohepatitis. On the other hand, hepatic inflammation might influence sleep disruption and sleepiness mediated by a liver-brain signalling mechanism such as cytokine translocation across the blood-brain barrier, similar to mechanism proposed for end-stage liver disease [36].

Human circadian sleep-wake regulation changes with age as a part of complex modifications of diurnal physiological systems accompanying ageing, such as hormonal and thermo-physiological alterations [37]. A methodological difficulty and limitation of this study was to match the age of healthy controls without evidence for the metabolic syndrome with patients diagnosed with NAFLD, given the prevalence of the metabolic syndrome is raising with age. We therefore accepted a numerically but not significantly higher age in NAFLD patients, but verified the findings in a subgroup analysis corrected for age. Conclusively, the results were independent of age, suggesting that the observed changes in sleep characteristics and timing of food-intake were disease-specific.

Sleep apnoea is a primary sleep disorder and has been described as a risk factor for NAFLD [28]. Interestingly it has recently been associated with the presence of inflammation (NASH) and fibrosis [38]. The data presented here detail sleep behaviour in NAFLD independent of its aetiologies, acknowledging a proportion of patients may have underlying OSA. In our NAFLD cohort, 7% of patients revealed an externally reported risk for OSA. To confirm the diagnosis of OSA, a specialist respiratory work-up including polysomnography in all patients with pathologic ESS or observed pauses in breathing would be required. However the consistence of results throughout the entire NAFLD cohort point towards a more general association of sleep disruption and NAFLD, rather than a subgroup effect alone, and suggest that sleep disturbances due to OSA as well as other aetiologies are related to NAFLD. A study supportive of our data previously showed OSA/ hypoxemia is prevalent in 60% of paediatric patients with NAFLD [39]. Here, most biochemical and histological parameters were comparable in NAFLD patients with and without OSA, except for fibrosis, which was more severe in the OSA group [39]. Additionally, a review of the literature reveals that the definitions used for OSA vary between studies and hereby limit the means to distinguish between the effects of sleep disruption secondary to OSA and independent of its aetiology on NAFLD.

In case the data presented here was corroborated by future studies detailing objective assessments of sleep disturbance in NAFLD, interventional studies targeting sleep-wake behaviour may be considered in patients with NAFLD.

The aims of interventional studies might be to shorten time to fall asleep, to prolong sleep duration and to avoid nocturnal food-intake. Preferably, such an intervention should be non-pharmacological and interfere with daily life as little as possible, such as sleep hygiene counselling, which is a treatment option for of affective disorders [40]. Another approach might be to study shifting sleep-wake timing using light therapy inducing artificial dawn and morning blue light [41,42]. In order to precisely document the changes in the sleep/activity pattern following interventional measures under study conditions, again objective parameters such as actigraphy, oscillation in physiological variables and hormones would need to be monitored.

Taken together, our data revealed specific quantitative and qualitative characteristics of sleep-disturbances in patients with NAFLD in addition to a shift of food-intake towards the night. The degree of daytime sleepiness was correlated to various parameters of disease severity. The findings therefore are suggestive for a pathophysiologic role of sleep and timing of food-intake in the pathogenesis of human NAFLD, they may endorse further studies validating sleep disturbances by objective methods, and potentially subsequent interventional studies evaluating re-alignment of sleep-wake behaviour as a treatment for NAFLD.

## Supporting Information

S1 Fig Sleep duration is associated with weight and BMI in patients with NAFLD. Sleep duration was negatively correlated with (A) weight (kg) and (B) BMI (kg/m^2^) in NAFLD. Spearman correlations.
(TIFF)

S2 Fig Quality of sleep impacts on negative and positive affect in patients with Non-alcoholic fatty liver disease (NAFLD) and positive affect is negatively correlated with liver function tests. **(**A) Sleep quality index (Pittsburgh Sleep Quality Index [PSQI]) was negatively correlated with positive affect scale (PANAS-PA) and (B) positively correlated with negative affect scale (PANAS-NA). (C) Positive affect was negatively correlated with ASAT and (D) ALAT. Spearman correlations.
(TIFF)

S1 File Eating habits questionnaire in English and German language. (PDF)

S1 Table Model validation identifying predictors of sleep duration. In order to identify predictors of sleep duration a model validation has been calculated. The model with the smallest prediction error was identified as the model predicting sleep duration best: lm(formula = sleep duration ~ scale(ESS) + group:scale(insulin) + scale(BMI) + scale(ASAT), data = feed). Predictors of shortened sleep duration were ESS, BMI and ASAT. A predictor of prolonged sleep duration was insulin, with differential effects for controls and NAFLD patients, respectively. Significance codes: ***, p<0.001, **, p<0.01, *, p<0.05,., p<0.1.
(DOCX)

S2 Table Biochemical parameters of Non-alcoholic fatty liver disease (NAFLD) patients and controls excluding subjects aged >66 years. NAFLD patients n = 37 and controls n = 22.
(DOCX)

S3 Table Sleep characteristics in patients with Non-alcoholic fatty liver disease (NAFLD) and controls excluding subjects aged >66 years. NAFLD n = 37 (simple steatosis n = 11; NASH n = 26) and controls n = 22. Hours (h). Minutes (min.). Munich Chronotype Questionnaire (MCTQ). Pittsburgh Sleep Quality Index (PSQI)). A score ≤ 5 is considered good sleep quality. Positive and negative affect scale (PANAS). Epworth Sleepiness scale (ESS). ESS score <10 is considered normal. #, significant difference Control vs. NAFLD; ‡, significant difference Controls vs. Steatosis; §, significant difference Control vs. NASH.
(DOCX)

S4 Table Eating habits in NAFLD patients and controls. NAFLD (n = 44); Controls (n = 22).
(DOCX)

S5 Table Daytime sleepiness is correlated with various biochemical parameters in non-cirrhotic NAFLD patients. The subgroup of non-cirrhotic patients was analysed (n = 35). Oral glucose tolerance tests were done in all non-diabetic subjects (n = 27). Spearman correlations.
(DOCX)
