# Supplementary figures and images for: Sleep Disruption and Daytime Sleepiness Correlating with Disease Severity and Insulin Resistance in Non-Alcoholic Fatty Liver Disease: A Comparison with Healthy Controls
Source: PLoS One. 2015 Nov 17;10(11):e0143293. doi: 10.1371/journal.pone.0143293 (PMC4648512; doi:10.1371/journal.pone.0143293)

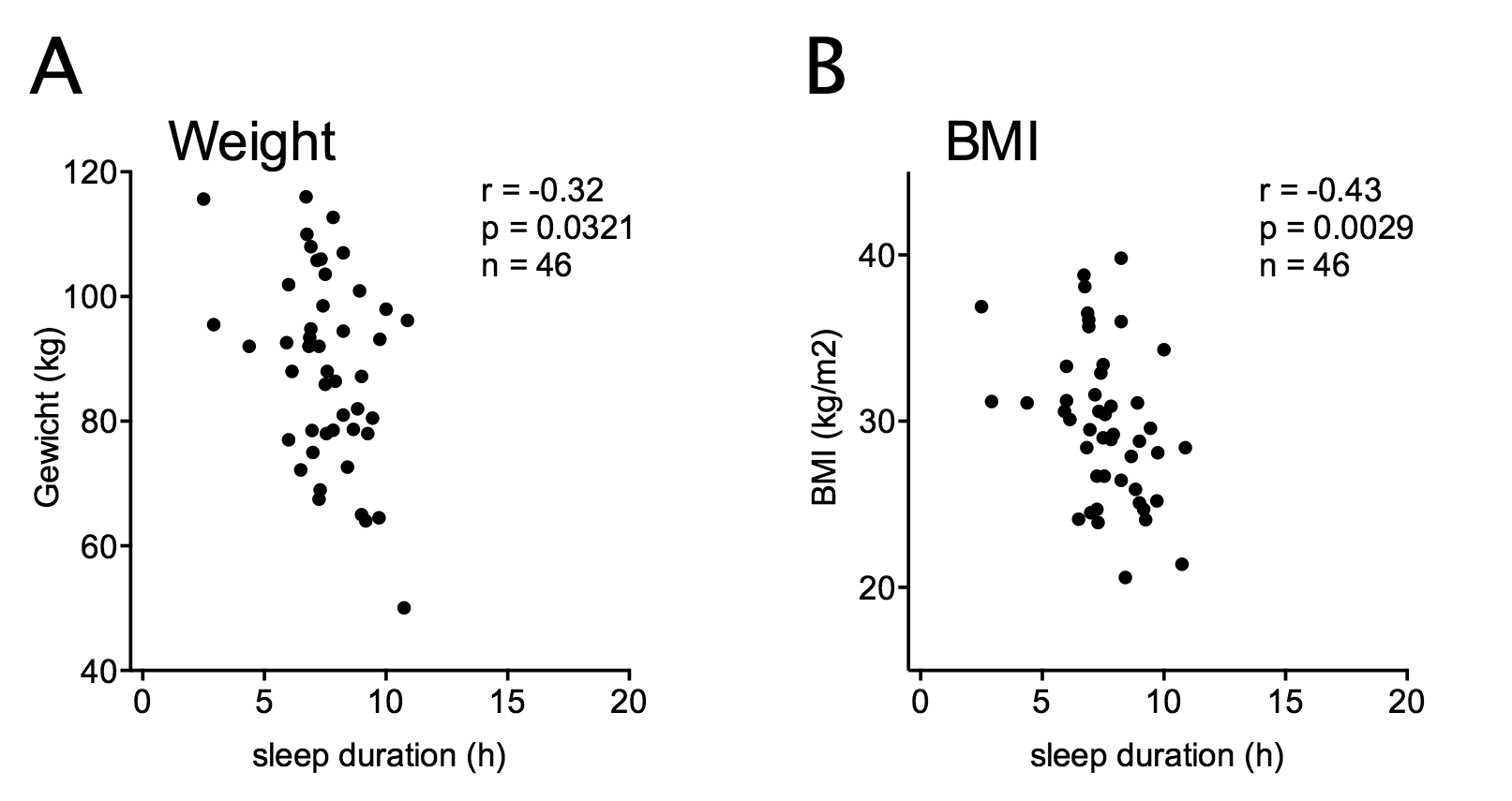

Supplement: S1 Fig — Sleep duration was negatively correlated with (A) weight (kg) and (B) BMI (kg/m2) in NAFLD. Spearman correlations. (TIFF) [file pone.0143293.s001.tiff]

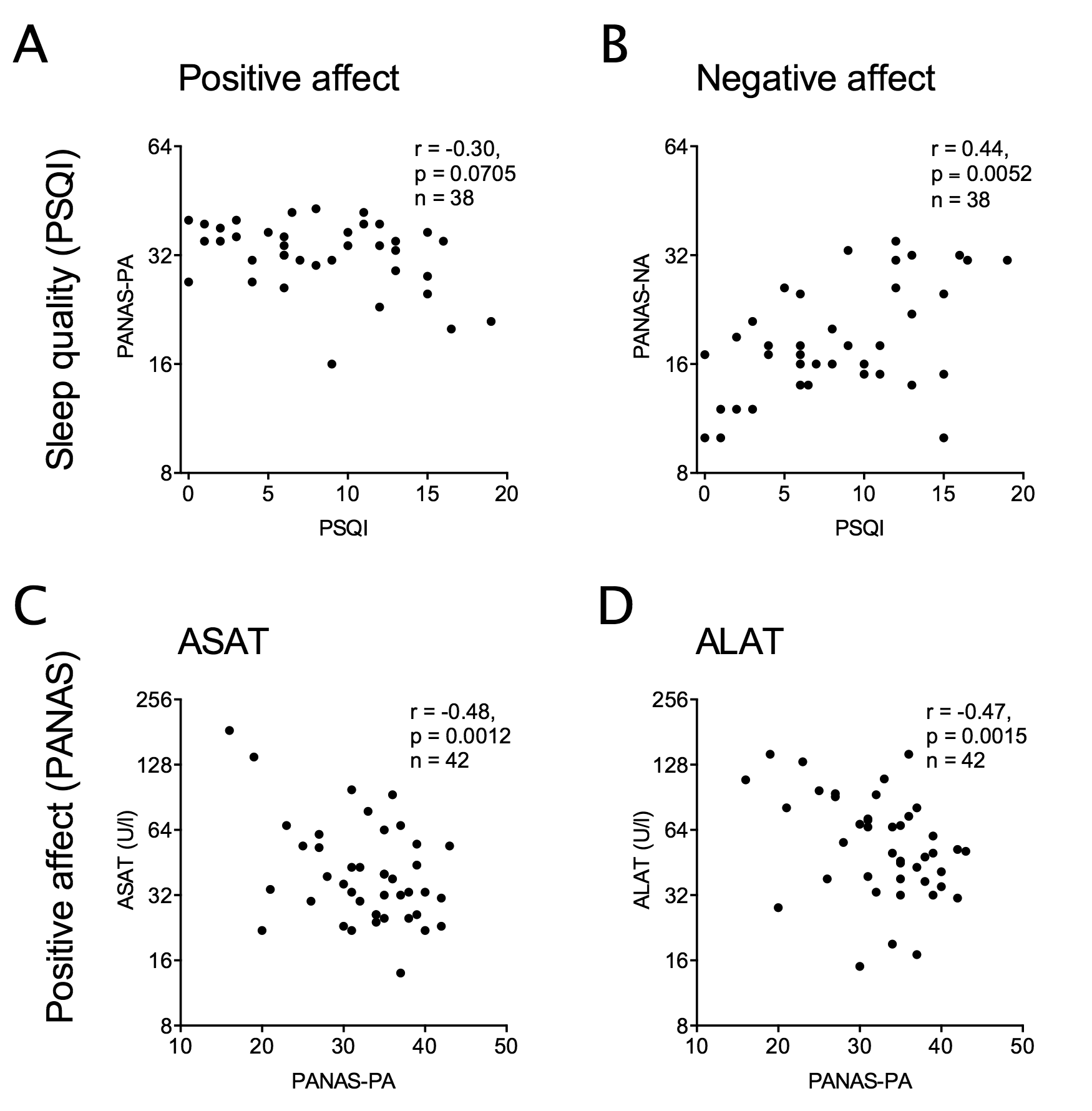

Supplement: S2 Fig — (A) Sleep quality index (Pittsburgh Sleep Quality Index [PSQI]) was negatively correlated with positive affect scale (PANAS-PA) and (B) positively correlated with negative affect scale (PANAS-NA). (C) Positive affect was negatively correlated with ASAT and (D) ALAT. Spearman correlations. (TIFF) [file pone.0143293.s002.tiff]
