# Supplementary material for: Sleep Disruption and Daytime Sleepiness Correlating with Disease Severity and Insulin Resistance in Non-Alcoholic Fatty Liver Disease: A Comparison with Healthy Controls
Source: PLoS One. 2015 Nov 17;10(11):e0143293. doi: 10.1371/journal.pone.0143293 (PMC4648512; doi:10.1371/journal.pone.0143293)
Supplement: S1 File — (PDF) [file pone.0143293.s003.pdf]

Eating habits questionnaire

Please answer the following questions, regarding your eating habits oft he previous 7 days. In case your eating habits during the previous 7 days have differed significantly from your habitual routine (because you may have been on holiday, you may have temporarily changed your work routine or a life event has occurred etc.), please regard the time before this period and highlight this (questions 22 and 23).

1. How many meals do you eat a day (breakfast, lunch, dinner, snacks)? Consider every fruit, every yoghurt, or a glass of milk etc. as a single snack.

…during the week

□ 1-2 meals a day
□ 3-4 meals a day
□ 5-6 meals a day
□ more than 6 meals a day

…at the weekend

□ 1-2 meals a day
□ 3-4 meals a day
□ 5-6 meals a day
□ more than 6 meals a day

2. At which times do you usually eat:

____ (time) □ meal □ snack
____ (time) □ meal □ snack
____ (time) □ meal □ snack
____ (time) □ meal □ snack
____ (time) □ meal □ snack
____ (time) □ meal □ snack

3. Please specify your meals for two specific days of the previous week:

Meal	Time	*Yesterday*	*Last Sunday*
breakfast			
lunch			
dinner			
snack I			
snack II			
snack III			
other			

4. How often do you have hot meals?

…during the week

- □ several times a day
- □ once daily
- □ more than 3 times a week
- □ less than 3 times a week
- □ never

...at the weekend

- □ several times a day
- □ once daily
- □ more than 3 times a week
- □ less than 3 times a week
- □ never

5. At which times do you have hot meals?

…during the week

- □ at lunchtime (____ [time])
- □ in the evening (____ [time])
- □ during the night (____ [time])
- □ other (____ [time])
- □ never

...at the weekend

- □ at lunchtime (____ [time])
- □ in the evening (____ [time])
- □ during the night (____ [time])
- □ other (____ [time])
- □ never

6. Which meals do you usually partake?

- □ breakfast
- □ lunch
- □ dinner
- □ snacks

7. Do you get up at nights to eat, or do you eat at night times because of sleeplessness?

- □ yes
- □ no

8. How much coffee/ tea do you have a day?

- □ none / less than one cup
- □ 1-2 cups
- □ 3-4 cups

□ 5-6 cups
□ more than 6 cups

9. How many alcoholic drinks do you have a week (examples for a standard drink: 1dL of wine, 3dL of beer, 2 cL of liquors)?
□ none or less than 1 standard drink a week
□ 1-3 standard drinks a week
□ 4-5 standard drinks a week
□ 6-7 standard drinks a week
□ 8-12 standard drinks a week
□ more than 12 standard drinks a week

10. How often do you eat in a restarant?
Your answer: ______________________________

11. In case you prepare your meals at home: do you serve single servings, or do you help yourself continuously until you feel sated?
□ single servings
□ repetitive serving to satiety

12. Do you watch out for healthy nutrition?
□ never
□ rarely
□ occasionally
□ usually

13. Are you mindful of your sensation of hunger?
□ never
□ rarely
□ occasionally
□ usually

14. Are you mindful of your sensation of satiety?
□ I realise when I become sated and stop eating
□ I realise when I become sated but continue eating
□ I rarely realise when I become sated and often eat too much
□ I arely realise when I become sated and eat according to the size of a standard serving

15. Do you eat when you feel stressed, unhappy, angry or bored?
□ never
□ rarely
□ occasionally

□ often

16. What type of diet you prefer?
    □ Swiss diet
    □ Mediterranean diet
    □ American diet
    □ Asian diet
    □ other ________________

17. Where do you use to buy food (you can choose more than one answer)
    □ on markets
    □ in supermarkets
    □ in small shops
    □ in organic food shops
    □ other ___________________

18. What type of food do you preferentially buy (you can choose more than one answer)
    □ pre-cooked foods
    □ fresh foods
    □ frozen foods
    □ canned foods
    □ other ___________________

19. Describe your physical activity during working hours:
    □ seated or standing position; few physical activities (e.g. office employee, watch maker, laboratory assistant, shop assistant, precision engineer, driver, assembly-line work)
    □ moderate physical activities (e.g. car mechanic, waiter, painter, electrician, nurse, housewife/househusband, gardener, postman)
    □ demanding physical activities (e.g. farmer, forester, builder, mason, carpenter, roofer, sportsman)

20. Describe your recreational physical activity:
    □ mainly seated activities; using motor vehicles for transport; using lift instead of stairs; no sports or just occasionally
    □ walking to work or taking the bicycle; using stairs instead of lifts; regular recreational exercise (at least 30 min. a day)
    □ regular physical activity in daily life (walking, cycling, climbing stairs etc.), endurance training (at least 20-60 min. 3 times weekly), strength training (at least twice a week)

21. Do you take your time to rest and relax?
    □ never
    □ rarely

- □ occasionally
- □ often

22. Did you eating habits during the previous 7 days differ significantly from your habitual routine of the previous month?
    - □ yes
    - □ no, the eating habits of the previous 7 days correspond to the previous month

23. Did you eating habits during the previous month differ significantly from your habitual routine of the previous year
    - □ yes
    - □ no, the eating habits of the previous month correspond to the previous year

24. Remarks

____
____
____
____

Fragebogen zu den Essgewohnheiten

Bitte beantworten Sie die Fragen, indem Sie Ihre Essgewohnheiten der vergangenen 7 Tage berücksichtigen. Sollte dieses sich grundlegend von Ihren sonstigen Essgewohnheiten unterscheiden (z.B. weil Sie in den Ferien waren, Sie aushilfsweise einer anderen Arbeit nachgegangen sind, ein besonderes Ereignis in Ihrem Leben eingetreten ist, etc.) so berücksichtigen Sie bitte das zeitintervall davor und kennzeichnen Sie dies (Fragen 22/23).

1. Wie viele Mahlzeiten pro Tag nehmen Sie zu sich? (Frühstück, Mittagessen, Abendessen, Zwischenmahlzeiten) Bereits eine Frucht, ein Glas Milch oder ein Jogurt gelten als Zwischenmahlzeit.

...während der Arbeitswoche:

□ 1-2 Mahlzeiten pro Tag
□ 3-4 Mahlzeiten pro Tag
□ 5-6 Mahlzeiten pro Tag
□ mehr als 6 Mahlzeiten pro Tag

...am Wochenende:

□ 1-2 Mahlzeiten pro Tag
□ 3-4 Mahlzeiten pro Tag
□ 5-6 Mahlzeiten pro Tag
□ mehr als 6 Mahlzeiten pro Tag

2. Zu welchen Tageszeiten nehmen Sie gewöhnlich Mahlzeiten ein:

____ Uhr □ Hauptmahlzeit □ Zwischenmahlzeit
____ Uhr □ Hauptmahlzeit □ Zwischenmahlzeit
____ Uhr □ Hauptmahlzeit □ Zwischenmahlzeit
____ Uhr □ Hauptmahlzeit □ Zwischenmahlzeit
____ Uhr □ Hauptmahlzeit □ Zwischenmahlzeit
____ Uhr □ Hauptmahlzeit □ Zwischenmahlzeit
____ Uhr □ Hauptmahlzeit □ Zwischenmahlzeit

3. Schreiben Sie Ihre Mahlzeiten von 2 Tagen der vergangenen Woche exemplarisch auf:

Mahlzeit	Uhrzeit	*Gestern*	*vergangenen Sonntag*
Frühstück			
Mittagessen			
Abendbrot			
Snack I			
Snack II			
Snack III			
Anderes			

4. Wie oft nehmen Sie warme Mahlzeiten zu sich?

...während der Arbeitswoche:

□ mehrmals täglich
□ täglich
□ mehr als 3x pro Woche
□ weniger als 3x pro Woche
□ nie

...am Wochenende:

□ mehrmals täglich
□ täglich
□ mehr als 3x pro Woche
□ weniger als 3x pro Woche
□ nie

5. Zu welcher Tageszeit nehmen Sie die warme Hauptmahlzeit zu sich?

...während der Arbeitswoche:

□ mittags (____ Uhr)
□ abends (____ Uhr)
□ nachts (____ Uhr)
□ zu einer anderen Tageszeit (____ Uhr)
□ nie

...am Wochenende:

□ mittags (____ Uhr)
□ abends (____ Uhr)
□ nachts (____ Uhr)
□ zu einer anderen Tageszeit (____ Uhr)
□ nie

6. Welche Mahlzeiten halten Sie gewöhnlich ein?

□ Frühstück
□ Mittagessen
□ Abendbrot
□ Snacks zwischendurch

7. Stehen Sie manchmal nachts auf um etwas zu essen oder essen Sie nachts weil Sie nicht schlafen können?

□ Ja
□ nein

8. Wie viel Kaffee/ schwarzen Tee trinken Sie pro Tag?

□ gar keinen / weniger als eine Tasse
□ 1-2 Tassen

- □ 3-4 Tassen
- □ 5-6 Tassen
- □ mehr als 6 Tassen

9. Wie viele Standarddrinks (alkoholische Getränke) trinken Sie pro Woche? Beispiele für Standarddrinks: 1 dl Wein, 3 dl Bier, 2 cl Spirituosen.

keine oder weniger als 1 Standarddrink pro Woche

- □ 1-3 Standarddrinks pro Woche
- □ 4-5 Standarddrinks pro Woche
- □ 6-7 Standarddrinks pro Woche
- □ 8-12 Standarddrinks pro Woche
- □ Mehr als 12 Standarddrinks pro Woche

10. Wie oft besuchen Sie im Durchschnitt Restaurants?

Ihre Antwort: ______________________________

11. Sofern Sie Ihre Hauptmahlzeiten zu Hause zubereiten: Bereiten Sie einzelne Portionen zu oder bedienen Sie sich mehrmals bis Sie sich satt fühlen?

- □ Portionen
- □ mehrmaliges Bedienen

12. Achten Sie auf eine gesunde Ernährung?

- □ Nie
- □ selten
- □ teilweise/gelegentlich
- □ meistens/immer

13. Achten Sie auf Ihr Hungergefühl?

- □ Nie
- □ selten
- □ teilweise/gelegentlich
- □ meistens/immer

14. Achten Sie auf Ihr Sättigungsgefühl?

- □ ich merke wann ich satt bin und höre dann auf zu essen
- □ ich merke wann ich satt bin, esse aber ab und zu trotzdem weiter
- □ ich spüre häufig nicht wann ich satt bin und überesse mich deshalb häufig
- □ ich spüre häufig nicht wann ich satt bin und orientiere mich an der Portionengrösse

15. Essen Sie, wenn Sie gestresst, verärgert, wütend sind oder sich langweilen?

- □ Nie
- □ selten
- □ teilweise/gelegentlich

□ meistens/immer

16. Welche Küche bevorzugen Sie? (mehrere Antworten möglich)
    □ Schweizer Küche
    □ Mediterrane Küche
    □ Amerikanische Küche
    □ Asiatische Küche
    □ Andere: ________________

17. Wo kaufen Sie gewöhnlich Ihr Essen? (mehrere Antworten möglich)
    □ auf Märkten
    □ im Supermarkt
    □ in kleinen Lebensmittelgeschäften
    □ im Bioladen
    □ Anderes: ___________________

18. Sie bevorzugen beim Einkauf eher... (mehrere Antworten möglich)
    □ Fertiggerichte
    □ frische Produkte
    □ Tiefkühlprodukte
    □ Konserven
    □ Anderes: ___________________

19. Wie sieht Ihre körperliche Aktivität im Berufsalltag aus?
    □ sitzende oder stehende Tätigkeit mit wenig Bewegung wie z.B. Büroangestellter, Uhrmacher, Laborant, Verkäufer, Feinmechaniker, Chauffeur, sitzende Fliessbandarbeit
    □ Tätigkeit mit mässiger körperlicher Bewegung wie z.B. Automechaniker, Serviceangestellter, Maler, Elektriker, Krankenpfleger, Hausfrau/Hausmann, Gärtner, Briefträger
    □ körperlich anstrengende Tätigkeit wie z.B. landwirtschaftliche Berufe, Förster, Waldarbeiter, Bauarbeiter, Maurer, Zimmermann, Dachdecker, Leistungssportler

20. Wie sieht Ihre körperliche Aktivität in der Freizeit aus?
    □ vorwiegend sitzende Tätigkeit, Fortbewegung hauptsächlich mit motorisierten Verkehrsmitteln, mehrheitlich Lift- anstatt Treppen-Benützung, kein oder nur sehr wenig Sport.
    □ Arbeitsweg zu Fuss oder mit Fahrrad, mehrheitlich Benützung der Treppen, regelmässiger Freizeitsport mit mittlerer Intensität (insgesamt eine halbe Stunde pro Tag).
    □ Einbau von Bewegung im Alltag (Arbeitsweg, Treppenbenützung, etc.) und mindestens drei Mal pro Woche Ausdauertraining (20 – 60 Minuten) und zwei Mal pro Woche Kraft- und Beweglichkeitstraining.

21. Nehmen Sie sich Zeit zum Ausruhen und Entspannen?

- □ Nie
- □ selten
- □ teilweise/gelegentlich
- □ meistens/immer

22. War Ihr Essverhalten in den vergangen 7 Tagen grundlegend anders als im vergangenen Monat?

- □ Ja
- □ Nein, die letzte Woche glich dem letzten Monat

23. War Ihr Essverhalten im vergangenen Monat grundlegend anders als im vergangenen Jahr?

- □ Ja
- □ Nein, der letzte Monat glich dem letzten Jahr

24. Bemerkungen

____

____

____

____
