# Supplementary material for: Sleep Disruption and Daytime Sleepiness Correlating with Disease Severity and Insulin Resistance in Non-Alcoholic Fatty Liver Disease: A Comparison with Healthy Controls
Source: PLoS One. 2015 Nov 17;10(11):e0143293. doi: 10.1371/journal.pone.0143293 (PMC4648512; doi:10.1371/journal.pone.0143293)
Supplement: S1 Table — In order to identify predictors of sleep duration a model validation has been calculated. The model with the smallest prediction error was identified as the model predicting sleep duration best: lm(formula = sleep duration ~ scale(ESS) + group:scale(insulin) + scale(BMI) + scale(ASAT), data = feed). Predictors of shortened sleep duration were ESS, BMI and ASAT. A predictor of prolonged sleep duration was insulin, with differential effects for controls and NAFLD patients, respectively. Significance codes: ***, p<0.001, **, p<0.01, *, p<0.05,., p<0.1. (DOCX) [file pone.0143293.s004.docx]

**Table S1**

|  | Estimate | Std. Error | T value | Pr(>\|t\|) |
| --- | --- | --- | --- | --- |
|  |  |  |  |  |
| (Intercept) | 6.5953 | 0.1673 | 39.415 | <2e-16 *** |
| Scale (ESS) | -0.3077 | 0.1693 | -1.818 | 0.0742 . |
| Scale (BMI) | -0.4725 | 0.1861 | -2.539 | 0.0138 * |
| Scale (ASAT) | -0.1896 | 0.1915 | -0.990 | 0.3262 |
| Group controls Scale (insulin) | 0.3652 | 0.6125 | 0.596 | 0.5533 |
| Group NAFLD patients Scale (insulin) | 0.3728 | 0.2012 | 1.853 | 0.0689 . |

Residual standard error: 1.233 on 59 degrees of freedom (DF)

Multiple R-squared:  0.1872, Adjusted R-squared:  0.1183

F-statistic: 2.717 on 5 and 59 DF,  p-value: 0.02821
