# Supplementary material for: Sleep Disruption and Daytime Sleepiness Correlating with Disease Severity and Insulin Resistance in Non-Alcoholic Fatty Liver Disease: A Comparison with Healthy Controls
Source: PLoS One. 2015 Nov 17;10(11):e0143293. doi: 10.1371/journal.pone.0143293 (PMC4648512; doi:10.1371/journal.pone.0143293)
Supplement: S2 Table — NAFLD patients n = 37 and controls n = 22. (DOCX) [file pone.0143293.s005.docx]

**Table S2**

|  | **NAFLD** | **Controls** | **NAFLD vs. Controls** |
| --- | --- | --- | --- |
|  |  |  |  |
| Age (years) | 48.0 ± 1.8 | 45.0 ±3.0 | *p=0.4867* |
| Weight (kg) | 89.6 ± 2.5 | 71.2± 1.9 | *p<0.0001* |
| BMI (kg/m^2^) | 30.1 ± 0.8 | 23.1 ± 0.4 | *p<0.0001* |
|  |  |  |  |
| Fasting glucose (mmol/l) | 6.2 ± 0.5 | 5.2 ± 0.1 | *ns* |
| Fasting insulin (mU/l) | 13.5 ± 2.3 | 5.0 ± 0.4 | *p=0.0090* |
| HOMA-IR | 4.1 ± 0.9 | 1.3 ± 0.1 | *p=0.0093* |
|  |  |  |  |
| ASAT (U/l) | 48.7 ± 5.7 | 26.2 ± 1.2 | *p=0.0006* |
| ALAT (U/l) | 65.5 ± 5.2 | 22.0 ± 1.7 | *p<0.0001* |
| GGT (U/l) | 103.2 ± 19.1 | 26.0 ± 4.5 | *p<0.0001* |
