# Supplementary material for: Sleep Disruption and Daytime Sleepiness Correlating with Disease Severity and Insulin Resistance in Non-Alcoholic Fatty Liver Disease: A Comparison with Healthy Controls
Source: PLoS One. 2015 Nov 17;10(11):e0143293. doi: 10.1371/journal.pone.0143293 (PMC4648512; doi:10.1371/journal.pone.0143293)
Supplement: S3 Table — NAFLD n = 37 (simple steatosis n = 11; NASH n = 26) and controls n = 22. Hours (h). Minutes (min.). Munich Chronotype Questionnaire (MCTQ). Pittsburgh Sleep Quality Index (PSQI)). A score ≤ 5 is considered good sleep quality. Positive and negative affect scale (PANAS). Epworth Sleepiness scale (ESS). ESS score <10 is considered normal. #, significant difference Control vs. NAFLD; ‡, significant difference Controls vs. Steatosis; §, significant difference Control vs. NASH. (DOCX) [file pone.0143293.s006.docx]

**Table S3**

|  | **NAFLD** |  |  | **Controls** |
| --- | --- | --- | --- | --- |
|  | **All** | **Steatosis** | **NASH** |  |
|  |  |  |  |  |
| Sleep duration (h) | 6.2 ± 0.2 # | 6.3 ± 0.4 ‡ | 6.1 ± 0.3 § | 7.2 ± 0.2 |
| Sleep latency (min.) | 27.9 ± 5.3 # | 18.45 ± 6.4 | 33.1 ± 7.3 § | 9.8 ± 2.0 |
| Bed time (h/24h) | 23.1 ± 0.2 | 23.2 ± 0.4 | 23.0 ± 0.3 | 22.9 ± 0.1 |
| MCTQ | 4.4 ± 0.3 | 4.4 ± 0.3 | 4.5 ± 0.4 | 4.9 ± 0.5 |
| PSQI | 8.2 ± 0.9 # | 6.3 ± 1.4 | 9.2 ± 1.1 § | 4.7 ± 0.8 |
| PANAS-NA | 19.3 ± 1.3 # | 16.3 ± 2.0 | 20.5 ± 1.5 § | 15.1 ± 1.1 |
| PANAS-PA | 32.3 ± 1.1 | 35.4 ± 1.5 | 31.2 ± 1.4 | 33.0 ± 1.4 |
| ESS | 7.1 ± 0.8 | 5.6 ± 0.9 | 7.7 ± 1.0 | 7.1 ± 0.8 |
