# Supplementary material for: Sleep Disruption and Daytime Sleepiness Correlating with Disease Severity and Insulin Resistance in Non-Alcoholic Fatty Liver Disease: A Comparison with Healthy Controls
Source: PLoS One. 2015 Nov 17;10(11):e0143293. doi: 10.1371/journal.pone.0143293 (PMC4648512; doi:10.1371/journal.pone.0143293)
Supplement: S4 Table — NAFLD (n = 44); Controls (n = 22). (DOCX) [file pone.0143293.s007.docx]

**Table S4**

|  | **NAFLD** | **Controls** | **NAFLD vs. Controls** |
| --- | --- | --- | --- |
|  |  |  |  |
| Frequency of meals (meals/d) | 1.9 ± 0.8 | 2.4 ± 0.2 | *p=0.0339* |
| Nocturnal meals (meals/d) | 0.16 ± 0.05 | 0.04 ± 0.04 | *ns* |
|  |  |  |  |
| Coffee consumption (cups/d) | 2.4 ± 0.2 | 1.7 ± 0.2 | *p=0.0016* |
| Alcohol consumption (drinks/d) | 1.4 ± 0.1 | 2.0 ± 0.2 | *p=0.0037* |
|  |  |  |  |
| Health & nutrition awareness (1-4) | 3.4 ± 1.0 | 3.7 ± 0.7 | *p=0.0447* |
| Hunger guided food intake (1-4) | 3.3 ± 0.1 | 3.8 ± 0.1 | *p=0.0187* |
| Satiety guided food intake (1-4) | 3.6 ± 0.1 | 3.5 ± 0.1 | *ns* |
| Stress related food intake (1-4) | 3.0 ± 0.2 | 2.6 ± 0.2 | *ns* |
|  |  |  |  |
| Physical activity, occupational (1-3) | 1.5 ± 0.1 | 1.6 ± 0.1 | *ns* |
| Physical activity, recreational (1-3) | 1.8 ± 0.1 | 2.1 ± 0.1 | *p=0.0524* |
