# Supplementary material for: Sleep Disruption and Daytime Sleepiness Correlating with Disease Severity and Insulin Resistance in Non-Alcoholic Fatty Liver Disease: A Comparison with Healthy Controls
Source: PLoS One. 2015 Nov 17;10(11):e0143293. doi: 10.1371/journal.pone.0143293 (PMC4648512; doi:10.1371/journal.pone.0143293)
Supplement: S5 Table — The subgroup of non-cirrhotic patients was analysed (n = 35). Oral glucose tolerance tests were done in all non-diabetic subjects (n = 27). Spearman correlations. (DOCX) [file pone.0143293.s008.docx]

**Table S5**

|  | **Spearman’s r** | **p-value** |
| --- | --- | --- |
| BMI (kg/m2) | 0.21 | 0.2156 |
| Fasting glucose (mmol/l) | 0.20 | 0.2237 |
| Fasting insulin (µU/ml) | 0.41 | 0.0169 |
| HOMA | 0.47 | 0.0056 |
| ASAT (U/l) | 0.50 | 0.0023 |
| ALAT (U/l) | 0.45 | 0.0064 |
| GGT (U/l) | 0.66 | <0.0001 |
| oGTT (Baseline) | 0.32 | 0.1015 |
| oGTT (30’) | 0.50 | 0.0077 |
| oGTT (60’) | 0.41 | 0.0375 |
| oGTT (90’) | 0.36 | 0.0665 |
